# Nerve transfers for the upper extremity function restoration

**DOI:** 10.1016/j.bas.2026.106050

**Published:** 2026-04-11

**Authors:** Lukas Rasulić, Andrija Savić, Milan Lepić

**Affiliations:** a Faculty of Medicine, University of Belgrade, Belgrade, Serbia; b Clinic for Neurosurgery, University Clinical Center of Serbia, Belgrade, Serbia; c Medical Faculty of the Military Medical Academy, University of Defence, Belgrade, Serbia; d Clinic for Neurosurgery, Military Medical Academy, Belgrade, Serbia

**Keywords:** Distal nerve transfer, Nerve transfer, Brachial plexus injury, Upper extremity reconstruction, Neurotization, Functional recovery

## Abstract

Nerve transfers have shifted from “salvage” procedures to a primary, biology-based strategy for restoring priority function after severe peripheral nerve injury, particularly brachial plexus injury. In this invited opinion, we summarize the historical roots of nerve transfer surgery and outline the modern rationale for distal coaptation - shortening regeneration distance, bypassing scarred zones, and directing axons toward functionally critical targets while preserving native biomechanics. We discuss practical donor-recipient selection principles (redundancy, axonal capacity, proximity, and synergy) and contrast proximal versus distal transfer concepts within contemporary reconstructive algorithms.

Using representative examples, we emphasize how distal transfers can be integrated with grafting, tendon transfers, or free muscle transfer in staged reconstructions. Based on our clinical experience across elbow, shoulder, and hand reinnervation, we highlight consistently high rates of functionally meaningful recovery and substantial improvements in patient-reported quality of life.

## Highlights

• Distal nerve transfers are a primary reconstructive strategy for restoring priority upper-limb function after major nerve injury.
• Practical donor–recipient selection (proximity, synergy, axon capacity) is central to predictable reinnervation and recovery.
• Standardized outcomes and multicenter prospective data are needed to strengthen evidence and refine modern algorithms.

## 1 Introduction

Peripheral nerve injuries, particularly brachial plexus injuries (BPI), remain among the most disabling conditions encountered in reconstructive neurosurgery. High-energy trauma, root avulsions, and proximal nerve disruptions frequently preclude direct repair or grafting, either due to the absence of viable proximal stumps or the excessive regeneration distance required for timely functional recovery. In such scenarios, nerve transfers have evolved from salvage procedures into a primary reconstructive strategy.

By rerouting axons of expendable donor nerves (or their fascicles) to a denervated but functionally critical recipient, nerve transfers enable biologic reinnervation while preserving native biomechanics. Since the pioneering work of Oberlin in 1994 (Oberlin et al., 1994),development of distal nerve transfers have fundamentally reshaped reconstructive algorithms for upper extremity nerve injuries (Bertelli et al., 2024).

## 2 Historical background

Although the field experienced rapid expansion after Oberlin's landmark description, the origins of nerve transfer date back to more than a century before.

After Laugier and Nélaton attempted first in human direct nerve sutures in 1864, and proved that reinnervation was surgically possible (Streppel et al., 2000), Sir Charles Ballance reported the first cross-nerve reanimation using the spinal accessory nerve (SAN) to reinnervate the facial nerve in 1895, establishing the principle of neurotization which would later become nerve transfer (Van de et al., 2009). The first application of nerve transfer for BPI was described by Tuttle in 1913, utilizing SAN and cervical plexus elements to restore priority motor functions (Tuttle, 1913).

Modern nerve transfers started to develop with intercostal nerve (ICN) transfers to the musculocutaneous nerve (MCN) for restoration of elbow flexion, independently described by Seddon and Yeoman in 1963, and spinal accessory nerve to suprascapular nerve transfer (SAN-SSN) for shoulder abduction and external rotation restoration, pioneered by Kotani et al., in 1972, which both served as a foundation of the reliable reinnervation in complete BPI ever since, paving the way for further innovation (Midha, 2004).

## 3 Nerve transfers concept & rationale

Nerve transfer is defined as a surgical coaptation of an expendable, intact donor nerve to a denervated but functionally more important recipient. The donor is carefully selected based on three principles (Oberlin et al., 1994): redundancy of function (its loss will be compensated by other muscles or overlapping territories) (Bertelli et al., 2024); adequate axonal capacity to support targeted reinnervation; and (Streppel et al., 2000) proximity to the target muscle to minimize regeneration distance (Woo et al., 2024).

The main objective of distal nerve transfers is to convert proximal nerve lesions into distal. By placing the coaptation close to the target muscle, distal transfers shorten axonal regeneration distance, accelerate reinnervation, and avoid irreversible muscle atrophy. Additional advantages include dissection in uninjured tissue planes, avoidance of scarred zones, and direct coaptation of the donor and recipient nerves minimizing the axonal loss at an additional suture line.

Donor nerves are preferably purely motor, expendable, synergistic with the recipient function, and, ideally, located near the target muscle (Brown et al., 2009). Although an axon donor-to-recipient ratio of approximately ≥0.7:1 has been associated with improved outcomes, reported axon counts vary widely and should be interpreted cautiously (Maniglio et al., 2025). Motor-to-motor matching remains ideal; however, cortical plasticity allows even antagonistic donors to be successfully retrained (Bertelli et al., 2024; Woo et al., 2024).

From a functional standpoint, reconstruction priorities generally follow a hierarchical order (Oberlin et al., 1994): restoration of elbow flexion - MCN (Bertelli et al., 2024) shoulder abduction, and stability - axillary (AXN) and SSN (Streppel et al., 2000); elbow and wrist extension - radial nerve (RN) (Van de et al., 2009); protective hand sensibility and hand and digits flexion - median nerve (MN); and finally (Tuttle, 1913) fine intrinsic hand functions - ulnar nerve (UN); which maximizes patient independence and safety.

The conceptual distinction between proximal and distal nerve transfers has further refined reconstruction algorithms, as proximal transfers exploit strong donor nerves near the plexus or spinal cord, whereas distal transfers shorten the regeneration distance by coapting closer to the target muscle. Seen as complementary strategies, they revamped surgical management of brachial plexus and peripheral nerve lesions to an unpreceded success (Bertelli et al., 2024; Woo et al., 2024).

## 4 Proximal nerve transfers

Proximal nerve transfers are essential when avulsions or long gaps make grafting impossible, and remain as a sole solution in complete brachial plexus palsy, where distal options are unavailable. Notable procedures include:

• SAN to SSN (shoulder abduction/external rotation).
• Intercostal nerves or phrenic nerve to MCN (elbow flexion).
• Contralateral C7 root to MN, RN and/or lower trunk (pan-plexus function).

Proximal transfers provide strong donor sources but involve long regeneration distances, delaying recovery and risking irreversible muscle denervation. Additionaly, dissection through unwelcome scarred tissue poses a significant challenge (Woo et al., 2024).

## 5 Distal nerve transfers

Distal nerve transfers involve coaptation of donor and recipient nerves near the target muscle, thereby dramatically shortening regeneration distance and enabling earlier functional recovery. They have become central to contemporary reconstructive strategies, particularly in partial BPI, isolated peripheral nerve lesions or when early restoration (supercharging) is indicated. Notable procedures include:

• *Oberlin procedure:* UN fascicle to biceps branch of the MCN
• *Double fascicular transfer:* Oberlin procedure + median fascicle to brachialis m.
• *Somsak procedure:* RN branch to the medial or long head of the triceps muscle (BLHT) to AXN
• *Tripple nerve transfer* (Double fascicular transfer + Somsak procedure)
• Pronator teres or branch to flexor carpi radialis (FCR) to posterior interosseous nerve (PIN).
• Anterior interosseous nerve (AIN) to deep motor branch of UN
• UN branch to abuctor digiti quinti to MN recurrent branch to the thenar
• Supercharge End-to-Side Nerve Transfer: AIN to UN

Donors are divided as distally and recipients as proximally as possible to allow direct coaptation without grafts. First signs of reinnervation typically appear within three months, functional recovery by one year, with continued improvement for several years (Bertelli et al., 2024). The refinement of techniques has broadened indications and improved outcomes across diverse injury patterns, and even in late transfers if the muscles are preserved, the results might surprise (Woo et al., 2024). End-to-side neurorrhaphy has emerged as an adjunctive strategy, particularly in situations with limited donor availability as it may serve as a “supercharging” technique to improve regeneration (Harnoncourt et al., 2025).

## 6 Outcomes

Across our single centre series addressing elbow, shoulder, and hand reinnervation, distal nerve transfers have consistently produced functionally meaningful recovery, objective gains in strength and range of motion, and substantial improvements in patient-reported quality of life (QoL).

For the restoration of priority functions, we have achieved the following: elbow function, the Oberlin transfer reliably restores flexion, while thoracodorsal-to-triceps transfer effectively reanimates extension, together achieving functional recovery in more than 95% of patients (Savić et al., 2026a). Shoulder reinnervation using SAN to SSN and BLHT-to-axillary nerve transfers yields high rates of satisfactory abduction and external rotation, with substantial pain reduction and QoL improvement (Savić et al., 2026d). In the hand, distal strategies tailored to individual nerves: radial (Savić et al., 2026b), median (Grujić et al., 2026), and ulnar (Savić et al., 2026c), consistently restored key motor functions (grasp, release, intrinsic control), translating into large QoL improvements (Table 1). Besides injury severity, and distal nerve transfer performed, the main factors influencing outcomes in our experience were time between injury and surgery and patient age, and these factors should not be underestimated.

**Table 1:** Summary of distal nerve transfers and outcomes across the series.

Target function	Distal nerve transfer(s)	Patients (n)	Key functional outcomes	Quality-of-life outcomes
Elbow flexion	Oberlin (ulnar fascicle → musculocutaneous);	24 + 2 mod.	Elbow flexion: M3–M5 in 95.8%; full ROM restored	PNSQoL from 26.7 preop. to 68.1 postop.
Elbow extension	Thoracodorsal → RN BLHT	7	Elbow extension: M3–M5 in 100%; full ROM restored	Poor/fair to very good–excellent
Shoulder abduction & external rotation	RN BLHT → axillary nerve	32	Abduction M3–M5 in 91.7%; mean ROM 103.6°	PNSQoL from 25.6 preop. to 61.7 postop.
			ER M3–M5 in 77.8%; mean ROM 92.5°	
	SAN → SSN	4	Abduction M3–M5 in 50%	
			ER M3–M5 in 0%	
Hand extension	Median FDS → ECRB;	9	100% M3–M5 wrist, finger, thumb extension; predominantly M4–M5	PNSQoL from 49 preop. to 78 postop. 88.9% excellent
	FCR → PIN			
Protective hand sensibility and hand and digits flexion	ECRB → AIN;	13	M4 finger flexion in 84.6%; grasp 22.8% → 62.8%; pinch 27.5% → 62.0%; Kapandji 4.2 → 7.5	PNSQoL from 41.3 preop. to 67.4 postop. majority excellent
	SUP → FDS;			
	ADM → thenar;			
	DDN → PDN			
Fine intrinsic hand functions	AIN to deep motor branch of UN	12	Overall satisfactory recovery 87.5%; grasp 42.3% → 82.5%; pinch 37.4% → 80.5%	Marked improvement in PNSQoL

## 7 Advantages, challenges and future directions

Nerve transfers have transitioned from salvage procedures to a central pillar of peripheral nerve reconstruction. Their success lies in biologic reinnervation, preservation of native biomechanics, and exploitation of cortical plasticity, enabling donor nerves to adopt new functional roles (Woo et al., 2024). By shortening regeneration distance, distal transfers offer earlier and more predictable recovery of priority functions (Bertelli et al., 2024).

Indications now extend beyond brachial plexus trauma to include isolated peripheral nerve lesions, tetraplegia, and selected central nervous system conditions. Nerve transfers can be integrated with grafting, tendon transfers, and free muscle transfers, allowing tailored, staged reconstructions (Bertelli et al., 2024; Woo et al., 2024). Less commonly used transfers allow for an effective treatment in less common injury patterns (e.g. Haninec & Kaiser's UN or MN fascicle transfer to the AXN in an upper brachial plexus palsy with concomitant radial nerve injury) (Haninec and Kaiser, 2012).

Nevertheless, prolonged timeline of recovery and limited donor availability remain a challenge. Emerging innovations (such as ultradistal intraneural transfers, bioengineered conduits, and neuroprosthetic integration) hold promise for further optimization.

The evidence base in peripheral nerve surgery remains dominated by single-center series, and consistency of results across different anatomical targets and donor-recipient combinations demands confirmation from coordinated multicentric collaboration, standardized outcome reporting, and prospective study designs capable of elevating the level of scientific evidence.

## 8 Conclusion

Proximal and distal nerve transfers are complementary strategies; however, distal transfers offer faster, more predictable, and functionally precise recovery. With growing evidence and expanding indications, nerve transfers (particularly distal techniques) are poised to surpass grafting and even direct repair as the preferred modality for upper extremity nerve reconstruction.

This series of papers on our rich historical foundation while sharing contemporary clinical experience. By highlighting modern techniques and excelent functional restoration, we aim to contribute to the continued refinement and promotion of nerve transfers as a defining reconstructive strategy in peripheral nerve surgery.

## Declaration of competing interest

The authors declare that they have no known competing financial interests or personal relationships that could have appeared to influence the work reported in this paper.
